# Telemedicine in Retinopathy of Prematurity: A Review of Current Evidence and Clinical Outcomes

**DOI:** 10.3390/children13101413

**Published:** 2026-10-15

**Authors:** Ilinca-Medeea Iftime, Andreea-Ramona Treteanu, Andreea Pușcașu, Mihai Craiu, Octaviana Cristea, Alina Angelica Ciolpan

**Affiliations:** 1 Faculty of Medicine, “Grigore T. Popa” University of Medicine and Pharmacy, 16 Universitatii Street, 700115 Iasi, Romania; 2 Department of Pediatrics, “Carol Davila” University of Medicine and Pharmacy, 050474 Bucharest, Romania; andreea-ramona.treteanu0720@stud.umfcd.ro (A.-R.T.);; 3 Center of Innovation and e-Health, “Carol Davila” University of Medicine and Pharmacy, 030167 Bucharest, Romania; 4 “Alessandrescu-Rusescu” National Institute for Mother and Child Health, Lacul Tei 120, 020395 Bucharest, Romania

**Keywords:** retinopathy of prematurity, telemedicine, tele-ROP, screening, management, diagnosis

## Abstract

**Highlights:**

**What are the main findings?**

- Telemedicine enables remote screening and monitoring of retinopathy of prematurity, with diagnostic performance comparable to conventional examinations for clinically significant and vision-threatening disease in selected settings.
- Artificial intelligence-assisted assessment and smartphone-based retinal imaging show potential to expand screening capacity and support the early identification of infants requiring specialist evaluation.

**What are the implications of the main findings?**

- Telemedicine may improve access to timely ROP screening, particularly in underserved and resource-limited settings with a shortage of trained specialists.
- Sustainable implementation requires adequate infrastructure, trained personnel, standardized screening and referral pathways, and appropriate ethical and legal safeguards.

**Abstract:**

**Background/Objectives**: Retinopathy of prematurity (ROP) is a leading cause of preventable childhood visual impairment, particularly in settings where access to specialized ophthalmologic care is limited. Telemedicine offers an alternative approach to ROP screening and monitoring through remote retinal imaging and expert interpretation. This narrative review aimed to evaluate the diagnostic performance, clinical benefits, implementation challenges, and future perspectives of telemedicine in ROP screening and management. **Methods**: A narrative literature review was conducted with consideration of the Scale for the Assessment of Narrative Review Articles (SANRA) criteria. PubMed, Scopus, and Google Scholar were searched in August and August 2026 for relevant publications from January 2000 to the search date. Two authors independently screened titles and abstracts, followed by full-text assessment according to predefined eligibility criteria. A total of 143 scientific articles and other high-quality sources were included in the final narrative synthesis. Evidence was evaluated qualitatively, without a formal risk-of-bias assessment. **Findings**: The reviewed literature indicates that telemedicine can expand access to ROP screening, facilitate remote specialist consultation, support longitudinal disease monitoring, and improve the triage of infants requiring urgent assessment. In selected settings, remote retinal image interpretation has demonstrated diagnostic performance comparable to conventional examinations for clinically significant and vision-threatening ROP. Artificial intelligence-based assessment and smartphone-based retinal imaging represent promising approaches to improving screening accessibility and workflow efficiency, particularly in resource-limited settings. However, implementation remains constrained by equipment costs, infrastructure limitations, image quality, workforce training requirements, and ethical and legal considerations. **Conclusions**: Telemedicine represents a promising complementary strategy for ROP screening and management, with the potential to improve access to timely specialist assessment and help prevent ROP-related visual impairment. Its successful integration into clinical practice requires sustainable infrastructure, standardized protocols, trained personnel, and reliable referral pathways that ensure timely in-person examination and treatment when indicated. Further research is needed to establish the effectiveness, cost-efficiency, and long-term sustainability of different telemedicine-based approaches across diverse healthcare settings.

## 1. Background

Retinopathy of prematurity (ROP) is a vasoproliferative retinal disorder that primarily affects preterm infants, particularly those with a birth weight < 1251 g [1,2,3,4], with a higher prevalence in underdeveloped or developing countries. In 2010, an estimated 184,700 preterm infants worldwide developed any stage of ROP [5,6]. Of these, an estimated 53,800 developed potentially vision-impairing severe disease requiring treatment, while approximately 20,000 experienced blindness or severe visual impairment attributable to ROP and a further 12,300 developed mild-to-moderate visual impairment [7]. The underlying mechanism of the disease is believed to involve a two-phase process: initially, delayed physiologic retinal vascularization results in a peripheral avascular retina, which is subsequently followed by vasoproliferation and pathologic neovascularization, which may extend into the vitreous [8,9,10].

Numerous studies have identified several risk factors associated with the development of severe forms of ROP, including low birth weight, slow postnatal growth, and non-Black race [11,12]. Clinical factors, such as the need for mechanical ventilation, severe chronic lung disease, and cardiorespiratory support, have also been strongly associated with disease severity [2,11]. Ophthalmologic findings at the initial examination are similarly predictive: an advanced stage of ROP at first presentation, a greater number of affected retinal quadrants, and the presence of retinal hemorrhages have all been linked to an increased risk of progression to severe disease [11,12,13,14,15]. Notably, approximately 8% of cases have been reported to progress to a severe form within a short interval of 2–3 weeks [12], underscoring the rapid and potentially aggressive nature of the condition in high-risk infants. Taken together, these findings highlight the importance of early identification and close monitoring of infants presenting with these risk factors. Prompt diagnosis and timely intervention are essential to prevent disease progression, minimize complications, and ultimately reduce the long-term burden and cost of care associated with ROP [16,17].

Regarding classification, ROP progresses from a demarcation line (stage 1) to a raised ridge (stage 2), followed by extraretinal fibrovascular proliferation (stage 3). Advanced disease includes partial retinal detachment (stage 4A) or involvement of the fovea (stage 4B) and culminates in total retinal detachment (stage 5), often with a funnel-shaped configuration obscuring the optic disc [18]. In addition to anatomical staging, ROP can be classified according to vascular activity, which reflects disease severity rather than the location or extent of retinal proliferation. This classification distinguishes plus disease, characterized by marked arterial tortuosity and venous dilation in the posterior retina, from pre-plus disease, which exhibits similar vascular changes that are less pronounced [19]. Treatment is recommended for Type 1 ROP, defined as zone I ROP at any stage with plus disease, zone I stage 3 ROP without plus disease, or zone II stage 2 or 3 ROP with plus disease [20]. Strabismus, amblyopia, and glaucoma represent the most frequently reported complications of ROP, although severe sequelae, such as retinal detachment and permanent visual impairment, may also occur [21].

Beyond its severe complications, ROP has emerged as a major global public health concern. We are currently facing the third ROP epidemic [22,23], characterized by a marked increase in the survival of preterm infants and, consequently, a dramatic rise in the number of diagnosed cases. As a result, ROP has become one of the leading causes of childhood blindness worldwide [24,25]. More recent data from the Global Burden of Disease Study 2019 indicate that approximately 2.17 million children and adolescents under 20 years of age were living with vision loss attributable to ROP in 2019, including more than 680,000 with complete blindness [26]. These estimates reflect the population-level burden of ROP-related visual impairment in individuals under 20 years of age in 2019 and are therefore not directly comparable with the 2010 estimates of annual ROP-related visual blindness and severe visual impairment described above.

Importantly, this form of blindness is largely preventable through timely screening, appropriate monitoring, and early intervention [22,23,24,25,27]. The prevalence of ROP and associated blindness varies regionally, reflecting differences in neonatal survival rates, availability of screening programs, and access to specialized care. In high-income countries, ROP incidence ranges from 17% to 30% among very preterm infants, whereas in some low- and middle-income countries, such as Nigeria, India, and Egypt, reported rates can exceed 40–50% in certain cohorts [28]. As the population of infants at risk for ROP continues to grow, while the number of trained ROP specialists remains relatively limited, it highlights the critical need for effective screening, timely diagnosis, and intervention, particularly in regions with limited resources, to prevent ROP-related blindness and reduce its long-term social and economic burden.

This imbalance has prompted the search for alternative solutions, ultimately leading to the adoption of telemedicine as a complementary screening strategy [16,29]. Telemedicine represents a significant advancement in healthcare systems, as it overcomes geographic barriers and enables patients in remote or underserved areas to access specialized care. Its implementation has contributed to reduced waiting times and lower transportation costs, while also improving the continuity of care. In addition, telemedicine facilitates the ongoing monitoring of chronic conditions through the use of portable imaging devices and enhances hospital workflow by enabling efficient digital triage of non-critical cases [30,31,32].

In the context of ROP, telemedicine involves the acquisition of retinal images, which are subsequently interpreted remotely by an expert ophthalmologist or trained non-physician [33,34,35,36]. This approach also allows for remote appointment scheduling, communication of diagnoses, treatment recommendations, and structured follow-up monitoring [37]. Importantly, telemedicine expands access to ROP screening, diagnosis, and management, particularly in regions with a limited availability of trained specialists [38,39].

This study aimed to evaluate the role of telemedicine in the screening, diagnosis, and management of ROP, with particular emphasis on the benefits and diagnostic performance, as well as associated implementation challenges, limitations, and future directions.

## 2. Methods

This narrative review was conducted with consideration of the SANRA (Scale for the Assessment of Narrative Review Articles) criteria [40]. A comprehensive literature search was performed in PubMed, Scopus, and Google Scholar to identify relevant evidence on the use of telemedicine in the screening, diagnosis, and management of retinopathy of prematurity (ROP). The initial search was conducted in August and August 2026 and included studies published from January 2000 to the date of the search.

The search strategy was developed around two main concepts: ROP and telemedicine. For PubMed, the following search formula was used:

(“Retinopathy of Prematurity”[MeSH Terms] OR “retinopathy of prematurity”[Title/Abstract]) AND (“Telemedicine”[MeSH Terms] OR “telemedicine”[Title/Abstract] OR “tele-ROP”[Title/Abstract] OR “teleophthalmology”[Title/Abstract] OR “telescreening”[Title/Abstract] OR “remote screening”[Title/Abstract]).

Equivalent search strategies were adapted to the indexing and search functions of Scopus and Google Scholar.

Studies were considered eligible if they were original research articles published in English between 2000 and the date of the search, had an available abstract and full text, and specifically addressed the use of telemedicine in the screening, diagnosis, monitoring, or management of ROP. Studies that were not directly relevant to the review topic, lacked sufficient methodological information, did not provide scientifically supported data, or were unavailable in full text were excluded. Conference abstracts without an associated full-text publication, case reports, letters, and editorials were also excluded.

After removing duplicate records, two authors independently screened the titles and abstracts. Potentially eligible articles subsequently underwent full-text assessment according to the predefined eligibility criteria. Disagreements regarding eligibility were resolved through discussion and consensus. Following the screening and eligibility assessment, a total of 143 scientific articles and other high-quality sources were included in the final narrative synthesis.

Given the narrative design of the review, the search and selection process was not intended to follow the fully systematic methodology of a systematic review, and no formal risk-of-bias assessment was performed. Nevertheless, the methodological robustness and clinical relevance of the included studies were considered qualitatively, taking into account study design, sample size, reference standards, and the reporting of diagnostic accuracy and clinical outcomes. Greater consideration was given to prospective and multicenter studies, systematic reviews, and clinical guidelines when synthesizing the available evidence.

## 3. Findings and Discussion

### 3.1. Role and Benefits of Telemedicine in ROP Screening and Management

Telemedicine has emerged as an important component of modern healthcare systems, facilitating access to medical expertise, across geographic distance. By reducing the need for travel, it may facilitate timely medical evaluation, expand screening capacity, and support earlier disease detection [40,41].

In the context of ROP screening, these benefits extend to both families and healthcare providers (Table 1). Parents can schedule and attend evaluations remotely, thereby saving time and financial resources while reducing stress and limiting infants’ exposure to transportation-related risks and potential infections [42,43,44]. For healthcare providers, telemedicine optimizes workflow efficiency by reducing the need for bedside travel and enabling trained personnel to capture retinal images, allowing specialists to focus on diagnostic interpretation and cases requiring urgent attention [16,33,35,45,46,47]. Several studies have also reported diagnostic performance comparable to conventional examination for specific ROP outcomes and under defined study conditions [48,49].

From a diagnostic perspective, telemedicine enables the acquisition and storage of retinal images that can be reviewed longitudinally to monitor disease progression and facilitate remote expert consultation, including second opinions, with accuracy comparable to the conventional examinations [46,49,50]. From a clinical management standpoint, telemedicine may improve the efficiency of screening and emergency triage, may reduce delays and healthcare costs, and may minimize the risks associated with transporting medically fragile infants or exposing them to infectious environments. These advantages are particularly relevant for patients in remote or underserved areas, where access to specialized ophthalmologic care may otherwise be limited [33,37,43,44,45,46,47].

**Table 1:** Key benefits of telemedicine interventions in ROP.

Category	Benefit	Description	References
Quality of care	Clinical performance	- results comparable to classic screening	[4,24,34,51,52,53,54,55]
	Follow-up and reevaluation	- digital image storage for disease progression tracking - facilitation of clinical reassessments	[35,46,49,50,51,56,57]
Healthcare management	Expansion of screening capacity	- increased number of infants examined - leveraged potential of the small number of experts in diagnosing ROP	[1,33,37,42,43,44,57]
	Emergency triage	- enables rapid remote assessment - prioritization of high-risk infants	[48]
	Personnel optimization	- image acquisition feasible by trained non-medical personnel - leverages the education and training of medical personnel	[35,39,45,51]
Patient safety	Transport risk mitigation	- minimizes risks associated with infant transport - lowers anxiety for caregivers during travel	[16,44]
	Epidemiological safety	- reduced infections and overcrowding - lower patient-doctor contact	[37,43,47,56]
Costs	Reduced financial and travel burden for caregivers	- reduced transportation costs - reduced indirect costs (lost wages, accommodations, childcare costs for siblings)	[16,37,42,56]
	Time savings	- reduces time spent by both doctor and patient for the consultation	[16,33]
	Minimized long-term negative outcomes	- prevents visual impairment in preterm infants - reduces risk of ROP-related and systemic complications - minimizes economic burden in settings lacking ROP screening specialists	[48,58]

#### 3.1.1. Improved Access to Care

The COVID-19 pandemic substantially restricted population mobility, with lockdowns and intercity travel limitations, reducing access to routine medical services [59,60]. As a consequence, many infants were temporarily deprived of timely ROP screening [61]. During this period, telemedicine experienced rapid expansion and demonstrated its clinical value as an alternative healthcare pathway [61,62]. Guo et al. reported that telemedicine consultations accounted for a markedly higher proportion of scheduled visits compared with face-to-face encounters (77.9% vs. 22.1%), and the rate of completed consultations was likewise greater for remote appointments (58.1% vs. 22.1%) [37]. Although several studies documented an overall decline in the number of infants screened for ROP during the pandemic, the implementation of telemedicine may have mitigated this impact by enabling continued access to screening services [5,6,63]. Consequently, the proportion of infants who were able to undergo ROP evaluation increased in parallel with the adoption of telemedical approaches [37,64].

In the context of the COVID-19 pandemic, telemedicine provided an alternative approach to overcome healthcare access limitations. Its implementation in ROP screening helped reduce overcrowding in medical facilities and may have contributed to lowering infection risk while allowing infants to remain at home or in local care settings [43,47]. This approach minimized exposure to external environments and avoided contact with other patients in waiting areas, while also limiting direct physician-patient interaction when not strictly necessary [37,47]. Moreover, given the global shortage of specialists trained in the diagnosis of ROP, telemedicine may expand access to screening, diagnosis, and clinical management for a larger number of infants, particularly in regions where ROP experts are unavailable [46,65,66].

#### 3.1.2. Timely Diagnosis and Intervention

The implementation of telemedicine is increasingly recognized as a potential valuable strategy for the early diagnosis of ROP, particularly in the context of a limited number of specialists trained in ROP relative to the growing population of at-risk infants [1,44]. By enabling remote retinal imaging and centralized expert review, telemedicine may expand screening capacity and facilitate access to specialized diagnostic assessment and clinical management for a larger number of infants. This is especially valuable for populations in underserved or geographically remote regions, where access to expert ophthalmologic care is often scarce [42,43,57].

Moreover, Richter et al. demonstrated that consultation times in telemedicine-based approaches are markedly shorter than those for traditional in-person evaluations [67]. This reduction in consultation time may allow more infants to be assessed within the same timeframe, reducing delays associated with long waiting lists and enabling earlier clinical intervention, which is crucial for preventing progression to severe or sight-threatening ROP [61].

Furthermore, telemedicine facilitates rapid triage in urgent cases. Infants identified with aggressive or sight-threatening ROP can be promptly referred to tertiary centers for immediate treatment, supporting timely therapeutic intervention and improving visual outcomes [47,68].

#### 3.1.3. High-Quality Retinal Imaging Technologies

Advances in telemedicine for ROP screening have led to the development of numerous devices capable of capturing high-quality images essential for accurate ROP diagnosis [69,70] (Table 2). The most widely used imaging device for ROP screening is the RetCam II/III system, which provides a wide field of view of approximately 130°, enabling high-quality retinal image acquisition [71]. Despite its diagnostic performance, the device is associated with substantial costs, limiting its availability in resource-constrained settings [72]. Nevertheless, lower-cost alternatives to wide-field cameras have been investigated. Lanzelotte et al. reported that the Pictor Plus camera, which offers a narrower 40° field of view and costs roughly 10% as much as wide-field systems, achieved diagnostic results comparable to those of experienced ophthalmologists for identifying plus and pre-plus disease [72]. Importantly, this device can be operated by trained non-medical personnel, thereby increasing accessibility in regions lacking ROP specialists, where telemedicine may represent the only feasible screening pathway [73]. For the Pictor Plus camera, Lanzelotte et al. reported a sensitivity of 63.2–65.8% and a specificity of 98.4–100% for pre-plus/plus disease [72]. A separate study reported 100% sensitivity and 74–93% specificity for the detection of clinically significant ROP, including type 1 ROP and pre-plus/plus disease [53]. Differences in diagnostic performance may reflect differences in study populations, outcome definitions, and grading methodology; therefore, these estimates should not be interpreted as directly comparable.

Similarly, Al-Abaiji et al. evaluated ROP screening using the ICON GO^®^ camera (Jasani Scientifics, Karachi, Pakistan) and found near-identical diagnostic outcomes when examinations were performed bedside compared with telemedicine-based assessment [35].

Additional studies have explored low-cost smartphone-based imaging systems combined with condensing lenses (+20D, +28D, or +40D) [9,23,50,58]. Although these setups provide a more limited field of view (approximately 30°), they have been shown to produce sufficiently high-quality retinal images for remote evaluation, thereby supporting screening, diagnosis, and management in settings with limited resources, where the absence of such technologies could result in preventable visual impairment or blindness [86]. The medical literature has reported several smartphone-based ophthalmoscopy tools, such as Peek Retina and D-Eye, which are capable of capturing high-quality retinal images in different ocular pathologies [86,87]. However, despite their demonstrated utility in general retinal imaging, evidence regarding their application in ROP screening remains limited in the current literature.

Standardized imaging protocols are essential for achieving optimal diagnostic reliability in telemedicine-based ROP screening [88]. Evidence indicates that a set of five retinal images of adequate quality is required. Reductions in either the image number or quality significantly decrease the likelihood of correctly identifying ROP, and signs of plus disease are reliably detected only when complete image sets are obtained [74,89].

Overall, recent technological advancements in telemedicine have introduced multiple imaging alternatives that combine high diagnostic utility with substantially lower costs. These advances of tele-ROP enhance accessibility in low-resource health systems and hold considerable potential for reducing ROP-related complications and preventing infant blindness worldwide [75,89,90].

#### 3.1.4. Cost-Effectiveness

The economic feasibility of telemedicine-assisted screening for ROP has been explored in several studies across different healthcare settings. A study conducted in Hungary evaluated the economic efficiency of bedside ROP screening using retinal imaging interpreted via telemedicine [91]. The authors demonstrated that, despite substantial initial implementation costs, the program proved cost-effective, with expenses fully amortized within the first four years [91]. Similarly, research from India described a mobile ROP screening model that operated without a permanently on-site ophthalmologist and was also found to be cost-effective, largely because screening expenditures were relatively low compared with the long-term economic and social burden associated with blindness [35]. In contrast, Isaac et al. compared the costs of telemedicine-based screening with traditional bedside examinations in Ontario and concluded that telemedicine may become more cost-effective primarily in settings with a high patient volume [29].

Based on previously published studies, telemedicine for ROP screening may be particularly cost-effective in settings with a substantial demand for specialized ophthalmologic expertise, particularly when the number of infants at risk is high and exceeds the capacity of available specialists to provide timely diagnosis and longitudinal monitoring [68,91]. In such contexts, telemedicine may help optimize resource allocation and expand access to care [92]. Conversely, in regions with relatively few infants requiring screening, telemedicine may be less economically advantageous, as its implementation and operational costs can surpass those associated with conventional bedside examinations performed directly by an ophthalmologist [93].

#### 3.1.5. Educational Value of Telemedicine in ROP

Telemedicine also plays a significant role in the education and training of ophthalmology trainees. By enabling exposure to a larger number and wider variety of cases, remote screening platforms allow learners to gain experience under the guidance of highly specialized ROP experts, thereby potentially improving diagnostic accuracy [93,94,95]. Patel et al. (2023) reported that, across countries with varying levels of development, incorrect diagnoses made by trainee ophthalmologists accounted for approximately half of all evaluated cases, highlighting the need for effective educational interventions [96]. In response to this challenge, Padhi et al. implemented a telemedicine system based on videoconferencing that enabled trainees to participate in real-time remote consultations, resulting in more effective learning [16]. Moreover, this approach facilitated ongoing physician-patient interaction, which may have contributed to greater parental acceptance by allowing direct communication with the specialist and real-time counseling. Similarly, Patel et al. (2017) assessed the effectiveness of tele-education by administering assessments before and after training and demonstrated substantial improvement in trainees’ diagnostic accuracy [97].

### 3.2. Effectiveness of Telemedicine Implementation in ROP

Efforts to determine the relative effectiveness of telemedicine versus traditional ophthalmoscopic examination for ROP diagnosis have produced mixed conclusions, and a consensus has yet to be reached.

Multiple international studies have evaluated the diagnostic performance of telemedicine-based screening compared with conventional binocular indirect ophthalmoscopy (BIO), with generally favorable findings regarding diagnostic performance for clinically significant ROP. Prospective multicenter studies conducted in North America have demonstrated that remote image-based grading achieves high sensitivity for detecting treatment-requiring ROP (TR-ROP), frequently exceeding 90%; specificity is also reported to be high in selected study settings. In particular, telemedicine has demonstrated strong performance in identifying plus disease and type 1 ROP, which are critical determinants of treatment initiation [52,53,54]. In contrast, Chiang et al. reported higher discriminatory performance for type 2 prethreshold or worse ROP and treatment-requiring ROP than for mild or worse disease, with areas under the ROC curve ranging from 0.905 to 0.946 and 0.941 to 0.968, respectively, compared with 0.747–0.896 for mild or worse ROP [55]. Although minor discrepancies have occasionally been reported in zone classification or subtle stage differentiation, these differences rarely alter clinical management decisions [4]. Furthermore, another study comparing telemedicine screening with conventional indirect ophthalmoscopy demonstrated that bedside ophthalmoscopy provides only a time-limited assessment without the possibility of image comparison, whereas telemedicine enables longitudinal evaluation through stored retinal images, allowing serial documentation and more comprehensive follow-up analysis [34]. Similarly, a systematic review analyzing over a dozen comparative studies has concluded that telemedicine provides diagnostic accuracy comparable to that of in-person ophthalmoscopic examinations, especially for clinically significant ROP [52]. Importantly, telemedicine offers the advantage of image documentation, enabling longitudinal comparisons, second opinions, and quality assurance features that are not inherently available during traditional bedside BIO examinations.

The diagnostic performance of telemedicine is closely dependent on the quality and characteristics of the retinal images obtained. Factors such as image quality, camera type, field of view, and the experience of the personnel acquiring the images may influence the ability to identify and correctly classify ROP. Similarly, grader expertise may affect the interpretation of subtle findings, particularly plus disease, disease stage, and zone classification [77,98]. Therefore, differences in diagnostic performance reported across studies should be interpreted in the context of the imaging technology, image acquisition protocol, and level of experience of both image acquisition personnel and remote graders [99].

Studies investigating the applications of telemedicine in ROP screening have expanded considerably in recent years, providing new insights into the potential for earlier diagnosis. Several studies have evaluated and compared different scoring systems designed to facilitate and refine the diagnosis of ROP through telemedicine-based assessment [97,100]. An example is the study of Xu et al., in which a retrospective analysis using data from the SUNDROP cohort was conducted, comparing the Telemedicine ROP Severity Score (TeleROP-SS) with the modified ROP Activity Scale (mROP-ActS) [98]. Among infants with treatment-requiring ROP, TeleROP-SS correctly identified 97.4% of the cases, whereas mROP-ActS achieved an accuracy of 66.7%. These findings suggest that TeleROP-SS may represent a more practical and reliable tool for monitoring disease progression in telemedicine-based screening programs.

On the other hand, Ossandón et al. compared telemedicine results obtained by non-expert evaluators with minimal training to those obtained by expert observers in diagnosing ROP [10]. The overall findings were broadly consistent across both groups, although diagnostic inaccuracies were more frequent among non-expert evaluators, especially in the assessment of plus disease and severe disease stages. This study highlighted the importance of expert evaluators in establishing accurate diagnoses and minimizing the risk of missed or misclassified disease. Nevertheless, by training non-medical workers to use a portable camera for ROP screening, high-quality images can be obtained in over 92–94% of cases, enabling experts to identify all severe cases of ROP [77]. These findings further support the importance of adequate training and image quality in telemedicine-based screening [85,99].

Collectively, international comparative data indicate that telemedicine can achieve high diagnostic performance for clinically significant and vision-threatening ROP in selected study settings and may provide additional workflow and documentation advantages, even if other studies have found that both methods are equally effective for detecting type 2 ROP or more severe disease [4,24].

### 3.3. Challenges and Limitations

#### 3.3.1. Costs of Implementation

The implementation of telemedicine in ROP screening is constrained by several limitations and barriers [99]. One of the primary challenges is limited reimbursement, which can prevent hospitals and clinics, particularly those in low-resource settings, from acquiring the specialized equipment necessary for effective telemedicine programs [38]. This issue is compounded by the high costs of retinal cameras, imaging devices, and other essential technology, which can hinder the consistent delivery of screening, timely diagnosis, and appropriate clinical management of infants at risk for ROP [5,33,36,39].

Economic evaluations of AI-based telemedicine in other ophthalmic conditions also provide relevant context for the potential financial implications of these technologies [101]. For example, Lin et al. reported that an AI-assisted telemedicine screening model for diabetic retinopathy achieved a modest 2.5% reduction in costs, alongside a 0.3% decrease in years spent without blindness and a 4% increase in quality-adjusted life years (QALYs) [102]. Although these findings were derived from an adult population with diabetic retinopathy and cannot be directly extrapolated to ROP, they illustrate the potential economic impact of AI-assisted telemedicine in ophthalmic screening.

Collectively, these financial constraints pose important challenges to the broad implementation and long-term sustainability of telemedicine-based ROP screening programs, particularly in low-resource settings, where access to specialized equipment and trained personnel is already limited [103].

#### 3.3.2. Infrastructure and Operational Gaps

Although telemedicine represents a highly innovative approach to ROP screening, its implementation is constrained by several technical and administrative challenges. A key technical limitation is the requirement for stable, high-speed Internet connections to transmit high-resolution retinal images [103,104]. In many settings, particularly in low-resource or rural hospitals, unreliable connectivity can delay image transfer, reduce screening efficiency, and compromise diagnosis timeliness. Furthermore, the quality of the acquired images can be affected by artifacts, improper focus, or suboptimal capture techniques, which may result in misinterpretation or diagnostic errors, ultimately jeopardizing patient outcomes [16,33].

From an administrative standpoint, many healthcare systems lack the necessary technological infrastructure to seamlessly integrate telemedicine-generated results into existing electronic medical records [77]. This gap creates workflow inefficiencies, increases administrative burdens, and may delay the communication of critical findings to the treating ophthalmologist or neonatal care team. Addressing these issues requires targeted investments in high-performance retinal imaging devices, reliable IT infrastructure, and secure data transmission systems that comply with regulatory standards for patient privacy and confidentiality [105,106].

Operationally, telemedicine programs depend heavily on the training of non-medical personnel responsible for image acquisition. Effective training programs are time-consuming and require significant financial and organizational resources [35,39,45]. Without adequate training, the quality of captured images may be insufficient, increasing the likelihood of misinterpretation by remote specialists and reducing the proportion of infants who are correctly diagnosed and treated in a timely manner [90]. In addition, challenges may also be encountered by ophthalmologists interpreting tele-ROP images. Braverman et al. evaluated ophthalmologists’ perspectives and experiences with tele-ROP and reported that only 40% of physicians who had used the system were satisfied with it [107]. The most commonly reported challenges were poor image quality and difficulty interpreting retinal photographs. These findings highlight that the effectiveness of tele-ROP depends not only on adequate training of personnel responsible for image acquisition but also on the quality of the images available for remote interpretation. Consequently, successful telemedicine-based ROP screening relies on appropriate staff training, high-quality image acquisition, and continuous quality assurance processes to support diagnostic accuracy and patient safety [108,109].

Evidence from established tele-ROP programs indicates that successful implementation requires not only reliable image acquisition and remote interpretation, but also clearly defined referral pathways and timely access to in-person ophthalmologic assessment and treatment when indicated [110,111,112,113,114,115]. Telemedicine serves as a screening and diagnostic support strategy and does not replace clinical examination or treatment when required. Consequently, the effectiveness of tele-ROP depends on the integration of remote screening with an appropriate referral system and timely access to definitive ophthalmologic care.

#### 3.3.3. Caregiver Acceptance and Satisfaction

For telemedicine tools used in ROP screening to be effective, acceptance by caregivers is essential for successful program implementation. Evidence from other ophthalmic settings may provide relevant contextual information regarding the acceptability of telemedicine [116]. Patel et al. (2022) investigated patient preferences for telemedicine versus face-to-face consultations during the COVID-19 pandemic and found that individuals with higher educational attainment and younger patients were more likely to prefer telemedicine and to rate virtual consultations positively [117]. However, this study evaluated adult patients with retinal disease and did not specifically assess parents or caregivers of infants undergoing ROP screening. Therefore, these findings should be considered indirect evidence regarding the acceptability of telemedicine rather than ROP-specific caregiver preferences.

In the context of ROP screening, several studies have reported favorable perceptions among parents and caregivers. Telemedicine is positively perceived from both clinical and parental perspectives. Das et al. demonstrated that modern non-contact retinal imaging techniques significantly reduce pain, stress, and physiological instability in premature infants, resulting in a more reassuring experience for parents [118]. Complementing these findings, Dhami et al. reported higher parental satisfaction when ROP screening was conducted via telemedicine, as parents perceived the procedure to be less invasive and less stressful for their child [119]. Additionally, the ability to view retinal images and receive clear explanations improved understanding of the condition, reduced anxiety, and increased confidence in diagnostic accuracy [119]. These findings suggest that, when appropriately implemented, telemedicine may be well accepted by parents and caregivers and may provide a reassuring alternative to conventional ROP screening.

Although telemedicine is a relatively recent addition to clinical practice, its increasing adoption across different ophthalmic settings suggests potential acceptability among patients and caregivers. However, acceptance may vary according to age, educational background, familiarity with digital technologies, and the specific clinical context. Therefore, findings from adult retinal disease populations should be distinguished from evidence specifically addressing parental or caregiver acceptance of telemedicine for ROP screening.

#### 3.3.4. Ethical and Legal Framework

In addition to obtaining informed parental consent, it is essential to provide clear explanations regarding how an infant’s retinal images are captured, transmitted, stored, and interpreted remotely. Similar considerations have been highlighted in studies and guidelines addressing telemedicine applications in other retinal and ocular conditions, underscoring the importance of transparent communication regarding the handling and remote interpretation of ophthalmic images [120]. Ensuring the confidentiality and security of patient data is critical, necessitating the use of secure transmission systems that comply with relevant national and international regulatory frameworks [121]. Furthermore, the adoption of standardized clinical protocols, comprehensive documentation practices, and clearly defined follow-up pathways is vital for safeguarding patient safety and maintaining accountability within telemedicine-based ROP screening programs [121,122].

### 3.4. Future Perspectives in Telemedicine-Based ROP

#### 3.4.1. Integration of Artificial Intelligence in Tele-ROP Systems for Predictive Screening and Clinical Decision Support

Since the onset of the COVID-19 pandemic, telemedicine has undergone substantial development, particularly through the integration of AI, which has enabled the creation of algorithms capable of assessing visual acuity and estimating ocular risk profiles [123]. One of the most promising future directions in tele-ROP screening is the integration of AI into diagnostic workflows. Machine learning and deep learning algorithms are increasingly capable of analyzing retinal images with high accuracy, particularly for detecting key features such as plus disease, vascular tortuosity, and early structural changes associated with treatment-requiring ROP [124]. Beyond simple image classification, emerging AI models are being developed to provide predictive risk stratification, integrating retinal imaging findings with clinical variables such as gestational age, birth weight, oxygen exposure, and systemic neonatal complications. Such predictive tools could allow clinicians to identify infants at highest risk for disease progression and prioritize them for closer monitoring or early intervention [78,123,124,125]. Furthermore, AI-driven telemedicine platforms may support automated triage systems, where retinal images captured in neonatal intensive care units (NICUs) are initially screened by algorithms. Only cases flagged as suspicious would require review by retinal specialists. This model could significantly reduce specialist workload and improve the scalability of screening programs, particularly in regions with limited ophthalmology resources [126].

Besides conventional tele-ROP, which involves remote interpretation of retinal images by an expert ophthalmologist, AI-based approaches can be broadly classified into AI-assisted and fully autonomous systems. AI-assisted tools support clinicians by analyzing retinal images and highlighting relevant disease features, while the final diagnosis and clinical decision remain the responsibility of the ophthalmologist. In contrast, fully autonomous systems independently analyze images and provide a diagnostic classification without routine expert interpretation. These approaches differ in their level of clinical autonomy, validation requirements, and need for clinical oversight [101].

Several studies have demonstrated that automated diagnostic systems, such as the ROPtool and i-ROP, can detect ROP from retinal images with accuracy comparable to that of expert clinicians, the latter with the additional capacity to identify pre-plus and plus disease [126,127,128]. Similarly, Brown et al. developed a deep convolutional neural network trained on 5511 retinal images to identify plus and pre-plus disease [129]. The algorithm achieved a sensitivity of 93% and a specificity of 94% for detecting plus disease and 100% sensitivity with 94% specificity for pre-plus disease. Notably, when evaluated on the same dataset, the fully automated system demonstrated superior performance compared to human expert graders. These technologies have the potential to strengthen future screening programs and expand access to care in regions where ROP specialists are scarce [130]. However, these conclusions are not universally supported because other studies have indicated that the implementation of such technologies may increase the number of examinations required compared to conventional ophthalmoscopy [38].

#### 3.4.2. Future Technologies to Improve Cost-Efficiency ROP Screening

The rapid integration of smartphone-based tools into the screening and diagnosis of ophthalmic diseases has been increasingly documented in recent years. In 2020, Aruljyothi et al. identified a total of 131 smartphone-based ophthalmic applications, the majority of which were designed for visual acuity assessment or eye relaxation exercises [22]. Although most of these applications were freely available (76%) and primarily targeted the general population (60%), their relatively low download rates suggest limited adoption, highlighting the need for improved dissemination strategies and greater user engagement to enhance their role in monitoring chronic ocular conditions. In contrast, more advanced smartphone-based technologies, such as fundus imaging systems, appear to offer greater clinical utility. As reported by Young et al., when integrated within telemedicine frameworks, these systems provide an efficient, accessible, and cost-effective approach to ophthalmic screening, particularly in resource-limited settings [5].

In response to the need for cost-effective screening strategies for ROP, emerging technologies have facilitated the development of smartphone-based ROP (S-ROP) solutions. Owing to their portability and relatively low cost, these systems offer a practical alternative to conventional imaging devices, particularly in low- and middle-income countries with under-resourced healthcare systems. By enabling wider access to screening, S-ROP has the potential to improve early detection and reduce the burden of vision-threatening complications among preterm infants [130,131].

S-ROP systems encompass a heterogeneous group of imaging solutions that can be broadly classified according to their technical design. One category includes smartphone devices used in different ocular pathologies coupled with indirect ophthalmoscopy adapters, such as clip-on systems (e.g., MII Ret Cam, CellScope Retina, oDocs Nun, and Paxos Scope) [50,132,133,134], as well as “do-it-yourself” (DIY) configurations. These systems typically rely on external condensing lenses and smartphone cameras to simulate indirect ophthalmoscopy. A second category comprises smartphone-based fundus camera systems, which are dedicated imaging devices integrated with smartphones, including platforms such as D-Eye (D-EYE, Truckee, CA, USA), Peek Retina (Peek Vision Foundation, London, UK), and the Phelcom Eyer^®^ camera (PHELCOM, Cambridge, MA, USA) [86,135,136]. Compared to adapter-based systems, these devices incorporate built-in optics and illumination, enabling more standardized image acquisition. Furthermore, they may present additional functionalities, such as capturing still retinal images or employing video-based acquisition with subsequent frame extraction. More recently, advances in AI have led to the development of S-ROP systems with integrated AI algorithms, enhancing image quality assessment and supporting more accurate and scalable diagnostic processes [137].

Numerous studies have evaluated the diagnostic performance of smartphone-based retinopathy of prematurity (S-ROP) systems in comparison with conventional screening modalities [22,69,137,138]. For instance, Young et al. compared smartphone-based fundus imaging systems [MII RetCam (MII Ret Cam Inc. Tamil Nadu, India) and the Keeler Monocular Indirect Ophthalmoscope (Keeler Ltd, Windsor, UK)] with standard wide-field digital fundus cameras, reporting a sensitivity of 100% and a specificity of 83.49% for the detection of clinically significant ROP [5]. The authors highlighted the advantages of smartphone-based imaging, particularly the satisfactory image quality and substantially lower cost, supporting its potential applicability in low- and middle-income countries. The integration of S-ROP systems with telemedicine platforms enables rapid transmission of images to remote specialists, reducing the need for patient and staff travel, particularly relevant in rural or resource-limited settings [68,130,139]. However, other studies have demonstrated that, although image quality was considered acceptable in up to 91.4% of examinations, it remains inferior to that obtained with contact wide-field imaging devices [70]. Additional limitations have been described by Wintergerst et al., including a smaller field of view and longer examination time, which may impact the efficiency and comprehensiveness of screening [23].

To address these technical challenges, future solutions in S-ROP involve the implementation of AI solutions that could support the entire process, from image acquisition to clinical decision-making. AI-enhanced S-ROP has the potential to improve disease screening performance, enable early detection of severe cases, provide standardized interpretation of findings, optimize the screening workflow by prioritizing urgent cases, and reduce the workload on an already overburdened healthcare system [5,138]. Recent studies from low- and middle-income countries indicate that combining S-ROP, AI, and telemedicine can substantially decrease per-patient costs, facilitate early detection of severe cases and prevent vision-threatening complications. In the future, more advanced AI algorithms could not only detect existing ROP but also predict disease progression, while integration with other neonatal health programs may allow multipurpose screening with a single device, maximizing efficiency and public health impact [140,141].

#### 3.4.3. Expansion of Global Screening Programs

The growing popularity and reported effectiveness of tele-ROP have led to the development of numerous national and international screening programs. These initiatives have achieved highly favorable outcomes, particularly by enabling the efficient evaluation of large populations of premature infants and significantly expanding access to timely diagnosis and care [110,111,112,113,114,115].

One of the earliest and largest telemedicine-based screening programs for ROP is the Karnataka Internet Assisted Diagnosis of Retinopathy of Prematurity (KIDROP), conducted in India [39,110]. In its initial phase, this program screened 7106 premature infants across 36 rural centers, identifying ROP in 22.39% of cases at various stages, with 3.57% requiring treatment. KIDROP has been widely recognized as a successful model for low- and middle-income countries facing a shortage of ROP specialists, addressing this gap through the use of portable telemedicine imaging systems that have demonstrated diagnostic performance comparable to standard clinical examinations. Similar telemedicine-based ROP screening programs have also been developed in high-income settings. In the United States, early initiatives included the Stanford University Network for Diagnosis of Retinopathy of Prematurity (SUNDROP), launched in 2005, followed by the e-ROP study, conducted between 2011 and 2013, both of which demonstrated the feasibility and accuracy of remote ROP diagnosis [142].

More recently, similar programs continue to evolve globally [93,143,144], with further evidence supporting the use of telemedicine in screening large populations of premature infants, particularly in regions with limited access to specialized care. Given their potential to improve screening coverage and address geographic and resource-related barriers, these tele-ROP models may further expansion and implementation in areas where screening coverage remains insufficient.

Despite these promising developments, the global expansion of tele-ROP programs remains influenced by challenges related to infrastructure, workforce training, data governance, and the need for standardized screening protocols. Addressing these barriers through coordinated global efforts and policy frameworks will be essential to ensure equitable and sustainable implementation [115].

## 4. Conclusions

Telemedicine represents an effective technological approach for the prevention of childhood blindness caused by retinopathy of prematurity, particularly in low-resource settings, where access to trained specialists is limited. Recent studies have shown that retinal images captured and interpreted remotely, either by experts or assisted by artificial intelligence, can yield diagnostic accuracy comparable to that of bedside ophthalmoscopic examinations. In addition, telemedicine offers several advantages, including expanded access to specialized care and potential cost reductions. Nevertheless, its large-scale implementation remains constrained by infrastructural limitations, the need for adequate training of personnel involved in image acquisition and system operation, and the necessity to strengthen the ethical and legal framework governing its use. Future efforts should focus on addressing these challenges to optimize the integration of telemedicine into clinical practice and ensure that all at-risk infants have timely access to effective screening and management.

## Figures and Tables

**Table 2:** Portable retinal cameras used in Tele-ROP.

Device	Type of Device	Field of View	Imaging Method	Portability	Cost	Diagnostic Performance	Key References
RetCam^®^	Wide-field camera	~130°	Contact	Semi-portable	High	- sensitivity of 100% and specificity of 97.5% in detecting prethreshold and threshold ROP [71] - reported cases of poor image quality (21%) [71] - sensitivity of 100% and specificity of 92% in detecting high-risk ROP [74]	[24,52,69,70,71,73,74,75,76,77]
Pictor^®^	Handheld camera	~40–45°	Non-contact	Highly portable	Medium	- sensitivity of 63.2–65.8% and specificity of 98.4–100% [72] - the mean time to acquisition of at least one satisfactory image of the posterior pole of each eye was 5 min [72] - 100% sensitivity for the pre-plus or plus disease and 74–93% specificity [53]	[53,72,73]
ICON GO^®^	Handheld camera	~100°	Contact	Portable	High	- near-identical diagnostic outcomes when examinations were performed bedside compared with telemedicine-based assessment [35]	[35,78]
Phoenix ICON^®^	Wide-field camera	~100°	Contact	Portable	High	- sensitivity of 82% and specificity of 77% for more than mild ROP. In type 1 ROP, sensitivity was 100% and specificity was 72.4% [79]	[79,80]
3Nethra Neo^®^	Wide-field camera	~120°	Contact	Portable	Medium	- sensitivity of 97–99% and specificity of 75–81% [81]	[81,82,83]
PanoCam^®^	Wide-field camera	~130°	Contact	Portable	Medium-High	- has intelligent software that assists the photographer in capturing the six - images per eye in predefined locations that are necessary to review and diagnose the newborn eye [84]	[84,85]

## Data Availability

No new data were created or analyzed in this study. Data sharing is not applicable to this article.

## Abbreviations

**ROP**	retinopathy of prematurity
**AI**	artificial intelligence
**QALYs**	quality-adjusted life years

## References

[1] Rosenblatt T.R., Ji M.H., Vail D., Ludwig C.A., Al-Moujahed A., Pasricha M.V., Callaway N.F., Kumm J., Moshfeghi D.M. (2021). Key factors in a rigorous longitudinal image-based assessment of retinopathy of prematurity. Sci. Rep..

[2] Keleş Şahï N.A., Uzun A. (2022). Impact of COVID-19 Outbreak on Retinopathy of Prematurity Screening. Middle Black Sea J. Health Sci..

[3] Wang S.K., Callaway N.F., Wallenstein M.B., Henderson M.T., Leng T., Moshfeghi D.M. (2015). SUNDROP: Six years of screening for retinopathy of prematurity with telemedicine. Can. J. Ophthalmol..

[4] Quinn G.E., Ells A., Capone A., Hubbard G.B., Daniel E., Hildebrand P.L., Ying G.-S. (2016). Analysis of Discrepancy Between Diagnostic Clinical Examination Findings and Corresponding Evaluation of Digital Images in the Telemedicine Approaches to Evaluating Acute-Phase Retinopathy of Prematurity Study. JAMA Ophthalmol..

[5] Young B.K., Cole E.D., Shah P.K., Ostmo S., Subramaniam P., Venkatapathy N., Tsai A.S.H., Coyner A.S., Gupta A., Singh P. (2023). Efficacy of Smartphone-Based Telescreening for Retinopathy of Prematurity With and Without Artificial Intelligence in India. JAMA Ophthalmol..

[6] Coyner A.S., Oh M.A., Shah P.K., Singh P., Ostmo S., Valikodath N.G., Cole E., Al-Khaled T., Bajimaya S., K.C. S. (2022). External Validation of a Retinopathy of Prematurity Screening Model Using Artificial Intelligence in 3 Low- and Middle-Income Populations. JAMA Ophthalmol..

[7] Kaur K., Mikes B.A. (2026). Retinopathy of Prematurity. StatPearls.

[8] Hartnett M.E., Penn J.S. (2012). Mechanisms and Management of Retinopathy of Prematurity. N Engl. J. Med..

[9] Ortiz A., Patiño S., Torres J., Mármol J., Serafin C., Dodhia R., Saidman G., Schbib V., Peña B., Monteoliva G. (2025). AI-Enabled Screening for Retinopathy of Prematurity in Low-Resource Settings. JAMA Netw. Open.

[10] Ossandón D., Zanolli M., López J.P., Stevenson R., Agurto R., Cartes C. (2015). Telemedicine correlation in retinopathy of prematurity between experts and non-expert observers. Arch. Soc. Esp. Oftalmol. Engl. Ed..

[11] Ying G.S., Quinn G.E., Wade K.C., Repka M.X., Baumritter A., Daniel E. (2015). Predictors for the Development of Referral-Warranted Retinopathy of Prematurity in the Telemedicine Approaches to Evaluating Acute-Phase Retinopathy of Prematurity (e-ROP) Study. JAMA Ophthalmol..

[12] Cheng Q.E., Quinn G.E., Daniel E., Baumritter A., Smith E., Ying G.S. (2020). Progression from preplus to plus disease in the Telemedicine Approaches to Evaluating Acute-Phase Retinopathy of Prematurity (e-ROP) Study: Incidence, timing, and predictors. J. Am. Assoc. Pediatr. Ophthalmol. Strabismus.

[13] Blazon M.N., Rezar-Dreindl S., Wassermann L., Neumayer T., Berger A., Stifter E. (2024). Retinopathy of Prematurity: Incidence, Risk Factors, and Treatment Outcomes in a Tertiary Care Center. J. Clin. Med..

[14] De Las Rivas Ramírez N., Luque Aranda G., Rius Díaz F., Pérez Frías F.J., Sánchez Tamayo T. (2022). Risk factors associated with Retinopathy of Prematurity development and progression. Sci. Rep..

[15] Di Pietro M., Decembrino N., Afflitto M.G., Malerba E., Avitabile T., Franco L.M., Longo A., Betta P. (2023). Risk factors in the development of retinopathy of prematurity: A 10-year retrospective study. Early Hum. Dev..

[16] Padhi T.R., Bhunia S., Das T., Nayak S., Jalan M., Rath S., Barik B., Ali H., Rani P.K., Routray D. (2024). Outcome of real-time telescreening for retinopathy of prematurity using videoconferencing in a community setting in Eastern India. Indian J. Ophthalmol..

[17] Kelkar J., Agashe S., Kelkar A., Khandekar R. (2017). Mobile unit for retinopathy of prematurity screening and management at urban Neonatal Intensive Care Units: Outcomes and impact assessment. Oman J. Ophthalmol..

[18] Chiang M.F., Quinn G.E., Fielder A.R., Ostmo S.R., Chan R.P., Berrocal A., Binenbaum G., Blair M., Campbell J.P., Capone A. (2021). International Classification of Retinopathy of Prematurity, Third Edition. Ophthalmology.

[19] Bai A. (2022). A New Era in the Screening and Diagnosis of Retinopathy of Prematurity: The Application of Artificial Intelligence. Master’s Thesis.

[20] (2003). Early Treatment For Retinopathy Of Prematurity Cooperative Group. Revised indications for the treatment of retinopathy of prematurity: Results of the early treatment for retinopathy of prematurity randomized trial. Arch. Ophthalmol..

[21] Hong E.H., Shin Y.U., Bae G.H., Choi Y.J., Ahn S.J., Kim I., Cho H. (2022). Ophthalmic complications in retinopathy of prematurity in the first decade of life in Korea using the national health insurance database. Sci. Rep..

[22] Aruljyothi L., Janakiraman A., Malligarjun B., Babu B.M. (2021). Smartphone applications in ophthalmology: A quantitative analysis. Indian J. Ophthalmol..

[23] Wintergerst M.W.M., Petrak M., Li J.Q., Larsen P.P., Berger M., Holz F.G., Finger R.P., Krohne T.U. (2019). Non-contact smartphone-based fundus imaging compared to conventional fundus imaging: A low-cost alternative for retinopathy of prematurity screening and documentation. Sci. Rep..

[24] Biten H., Redd T.K., Moleta C., Campbell J.P., Ostmo S., Jonas K., Chan R.V.P., Chiang M.F. (2018). Diagnostic Accuracy of Ophthalmoscopy vs Telemedicine in Examinations for Retinopathy of Prematurity. JAMA Ophthalmol..

[25] Quinn G.E., Ying G.S., Repka M.X., Siatkowski R.M., Hoffman R., Mills M.D., Morrison D., Daniel E., Baumritter A., Hildebrand P.L. (2016). Timely implementation of a retinopathy of prematurity telemedicine system. J. Am. Assoc. Pediatr. Ophthalmol. Strabismus.

[26] Wang S., Liu J., Zhang X., Liu Y., Li J., Wang H., Luo X., Liu S., Liu L., Zhang J. (2024). Global, regional and national burden of retinopathy of prematurity in childhood and adolescence: A spatiotemporal analysis based on the Global Burden of Disease Study 2019. BMJ Paediatr. Open.

[27] Rogers D.L., Bremer D.L., Fellows R.R., Baumritter A., Daniel E., Pastilli M., Ying G.-S., Quinn G.E. (2017). Comparison of strategies for grading retinal images of premature infants for referral warranted retinopathy of prematurity. J. Am. Assoc. Pediatr. Ophthalmol. Strabismus.

[28] Nair A., El Ballushi R., Anklesaria B.Z., Kamali M., Talat M., Watts T. (2022). A Review on the Incidence and Related Risk Factors of Retinopathy of Prematurity Across Various Countries. Cureus.

[29] Isaac M., Isaranuwatchai W., Tehrani N. (2018). Cost analysis of remote telemedicine screening for retinopathy of prematurity. Can. J. Ophthalmol..

[30] Ezeamii V.C., Okobi O.E., Wambai-Sani H., Perera G.S., Zaynieva S., Okonkwo C.C., Ohaiba M.M., William-Enemali P.C., Obodo O.R., Obiefuna N.G. (2024). Revolutionizing Healthcare: How Telemedicine Is Improving Patient Outcomes and Expanding Access to Care. Cureus.

[31] Haleem A., Javaid M., Singh R.P., Suman R. (2021). Telemedicine for healthcare: Capabilities, features, barriers, and applications. Sens. Int..

[32] Stoltzfus M., Kaur A., Chawla A., Gupta V., Anamika F.N.U., Jain R. (2023). The role of telemedicine in healthcare: An overview and update. Egypt J. Intern Med..

[33] Bao Y., Ming W.K., Mou Z.W., Kong Q.H., Li A., Yuan T.F., Mi X.S. (2021). Current Application of Digital Diagnosing Systems for Retinopathy of Prematurity. Comput Methods Programs Biomed..

[34] Rosenblatt T.R., Ghoraba H.H., Ji M.H., Baumal C.R., Berrocal A.M., Besirli C.G., Drenser K.A., Ells A.L., Harper C.A., Hubbard G.B. (2025). Longitudinal Assessment of Retinopathy of Prematurity (LONGROP) Study: Impacts of Viewing Time and Ability to Compare on Detection of Change. Am. J. Ophthalmol..

[35] Al-Abaiji H.A., Bangsgaard R., Kofod M., Faber C., Larsen A.-C., Brost A.G., Slidsborg C., Klemp K., Breindahl M., de la Cour M.D. (2024). Evaluating the Feasibility of a Telescreening Program for Retinopathy of Prematurity (ROP) in Denmark. J. Pers. Med..

[36] Kelkar J., Kelkar A., Sharma S., Dewani J. (2017). A mobile team for screening of retinopathy of prematurity in India: Cost—Effectiveness, outcomes, and impact assessment. Taiwan J. Ophthalmol..

[37] Guo Z., Ma N., Wu Y., Yuan H., Luo W., Zeng L., Jie H., Li S. (2021). The safety and feasibility of the screening for retinopathy of prematurity assisted by telemedicine network during COVID-19 pandemic in Wuhan, China. BMC Ophthalmol..

[38] Morrison S.L., Dukhovny D., Chan R.V.P., Chiang M.F., Campbell J.P. (2022). Cost-effectiveness of Artificial Intelligence–Based Retinopathy of Prematurity Screening. JAMA Ophthalmol..

[39] Vinekar A., Mangalesh S., Jayadev C., Gilbert C., Dogra M., Shetty B. (2017). Impact of expansion of telemedicine screening for retinopathy of prematurity in India. Indian J. Ophthalmol..

[40] Baethge C., Goldbeck-Wood S., Mertens S. (2019). SANRA-a scale for the quality assessment of narrative review articles. Res. Integr. Peer Rev..

[41] Anawade P.A., Sharma D., Gahane S. (2024). A Comprehensive Review on Exploring the Impact of Telemedicine on Healthcare Accessibility. Cureus.

[42] Golan-Cohen A., Vinker S., Merzon E., Green I., Israel A. (2025). The impact of telemedicine on physician time invested in primary care: A large population-based descriptive study. Prim. Health Care Res. Dev..

[43] Jeyabal P., Tan C., Koh V. (2020). Survey of General Practitioners on Tele-Ophthalmology Practice in Singapore. Ann. Acad. Med. Singap..

[44] Wong J.K.W., Zhu M.M., Lam J.C.H., Leung K.M.-K., Lian J.X., Lam C.L.-K., Shih K.C., Lai J.S.-M. (2022). Prospective Comparative Study Investigating Agreement between Tele-Ophthalmology and Face-to-face Consultations in Patients Presenting with Chronic Visual Loss. Ophthalmol. Ther..

[45] Katsan S., Adakhovska A., Pasyechnikova N. (2024). Mobile model of ophthalmological care for children with retinopathy of prematurity in the southern region of Ukraine. Klin. Ocz..

[46] Gurwin J., Tomlinson L.A., Quinn G.E., Ying G.-S., Baumritter A., Binenbaum G., for the Postnatal Growth and Retinopathy of Prematurity (G-ROP) Study Group and the Telemedicine Approaches to Evaluating Acute-Phase Retinopathy of Prematurity (e-ROP) Cooperative Group (2017). A Tiered Approach to Retinopathy of Prematurity Screening (TARP) Using a Weight Gain Predictive Model and a Telemedicine System. JAMA Ophthalmol..

[47] Bain L., Kristensen-Cabrera A., Lee H. (2018). A Qualitative Analysis of Challenges and Successes in Retinopathy of Prematurity Screening. Am. J. Perinatol. Rep..

[48] Deshmukh A., Badakere A., Sheth J., Bhate M., Kulkarni S., Kekunnaya R. (2020). Pivoting to teleconsultation for paediatric ophthalmology and strabismus: Our experience during COVID-19 times. Indian J. Ophthalmol..

[49] Mohammadi S.F., Rahban A., Darabeigi S., Salimi N., Farahani A., Lashay A., Alinia C. (2021). Cost-effectiveness analysis of tele-retinopathy of prematurity screening in Iran. Int. J. Ophthalmol..

[50] Ossandón D., Zanolli M., Stevenson R., Agurto R., Ortiz P., Dotan G. (2018). A national telemedicine network for retinopathy of prematurity screening. J. Am. Assoc. Pediatr. Ophthalmol. Strabismus.

[51] Lekha T., Ramesh S., Sharma A., Abinaya G. (2019). MII RetCam assisted smartphone-based fundus imaging for retinopathy of prematurity. Indian J. Ophthalmol..

[52] Li W., Cheung R., Malvankar-Mehta M.S. (2022). Comparing the diagnostic accuracy of telemedicine utilization versus in-person clinical examination for retinopathy of prematurity in premature infants: A systematic review. J. Am. Assoc. Pediatr. Ophthalmol. Strabismus.

[53] Brady C.J., D’Amico S., Campbell J.P. (2020). Telemedicine for Retinopathy of Prematurity. Telemed. E-Health.

[54] Bowe T., Ung C., Campbell J.P., Yonekawa Y. (2019). Telemedicine for Retinopathy of Prematurity in 2020. J. Vitreoretin Dis..

[55] Chiang M.F., Starren J., E Du Y., Keenan J.D., Schiff W.M., Barile G.R., Li J., A Johnson R., Hess D.J., Flynn J.T. (2006). Remote image-based retinopathy of prematurity diagnosis: A receiver operating characteristic analysis of accuracy. Br. J. Ophthalmol..

[56] Redd T.K., Campbell J.P., Brown J.M., Kim S.J., Ostmo S., Chan R.V.P., Dy J., Erdogmus D., Ioannidis S., Kalpathy-Cramer J. (2019). Evaluation of a deep learning image assessment system for detecting severe retinopathy of prematurity. Br. J. Ophthalmol..

[57] Li J.P.O., Liu H., Ting D.S.J., Jeon S., Chan R.V.P., Kim J.E., Sim D.A., Thomas P.B.M., Lin H., Chen Y. (2021). Digital technology, tele-medicine and artificial intelligence in ophthalmology: A global perspective. Prog. Retin Eye Res..

[58] Asturias A.L., Gilbert C., Silva J.C., Quinn G.E. (2022). Implementation of telemedicine screening for retinopathy of prematurity in rural areas in Guatemala. J. Am. Assoc. Pediatr. Ophthalmol. Strabismus.

[59] Choudhary R.A., Hashmi S., Tayyab H. (2023). Smartphone-based fundus imaging for evaluation of Retinopathy of Prematurity in a low-income country: A pilot study. Pak. J. Med. Sci..

[60] Filip R., Gheorghita Puscaselu R., Anchidin-Norocel L., Dimian M., Savage W.K. (2022). Global Challenges to Public Health Care Systems during the COVID-19 Pandemic: A Review of Pandemic Measures and Problems. J. Pers. Med..

[61] Sood S., Naguib M.M., Portney D.S., Besirli C.G., Martin C.A., Harper C.A., Fernandez M.P., Berrocal A.M., Quiram P.A., Belin P. (2023). The impact of the COVID-19 lockdown on retinopathy of prematurity screening and management in the United States: A multicenter study. J. Am. Assoc. Pediatr. Ophthalmol. Strabismus.

[62] Johnson C., Taff K., Lee B.R., Montalbano A. (2020). The rapid increase in telemedicine visits during COVID-19. Patient Exp. J..

[63] Shaver J. (2022). The State of Telehealth Before and After the COVID-19 Pandemic. Prim. Care Clin. Off. Pract..

[64] Chakraborty S., Sheth J. (2023). Impact of COVID-19 Pandemic on Retinopathy of Prematurity Services in the Indian Public Healthcare System. Clin. Ophthalmol..

[65] Ravindran M., Segi A., Mohideen S., Allapitchai F., Rengappa R. (2021). Impact of teleophthalmology during COVID-19 lockdown in a tertiary care center in South India. Indian J. Ophthalmol..

[66] Puri S., Sarpal S., Ashat M. (2014). Screening of retinopathy of prematurity: A neglected public health issue. Ann. Med. Health Sci. Res..

[67] Richter G.M., Sun G., Lee T.C., Chan R.P., Flynn J.T., Starren J., Chiang M.F. (2009). Speed of telemedicine vs ophthalmoscopy for retinopathy of prematurity diagnosis. Am. J. Ophthalmol..

[68] Ayesiga I., Magala P., Ediamu T.D., El Diwany H., Yeung I.L., Wasomoka A., Muwonge H., Musoke P., Musiime E., Tumwesigye A. (2025). Do neonatal intensive care unit (NICU) health workers know about retinopathy of prematurity (ROP)? A qualitative study at a Regional Referral Hospital in Uganda. BMJ Paediatr. Open.

[69] Antaki F., Bachour K., Kim T.N., Qian C.X. (2020). The Role of Telemedicine to Alleviate an Increasingly Burdened Healthcare System: Retinopathy of Prematurity. Ophthalmol. Ther..

[70] Lin J.Y., Kang E.Y.C., Banker A.S., Chen K.-J., Hwang Y.-S., Lai C.-C., Huang J.-L., Wu W.-C. (2022). Comparison of RetCam and Smartphone-Based Photography for Retinopathy of Prematurity Screening. Diagnostics.

[71] Wu C., Petersen R.A., VanderVeen D.K. (2006). RetCam Imaging for Retinopathy of Prematurity Screening. J. Am. Assoc. Pediatr. Ophthalmol. Strabismus.

[72] Lanzelotte V., Vieira A.A., Monteiro Fonseca A.B., Eduardo Da Silva J., Gonet B., Bueno A.C. (2022). ROP screening with the Pictor Plus camera: A telemedicine solution for developing countries. J. Am. Assoc. Pediatr. Ophthalmol. Strabismus.

[73] Prakalapakorn S.G., Wallace D.K., Freedman S.F. (2014). Retinal imaging in premature infants using the Pictor noncontact digital camera. J. Am. Assoc. Pediatr. Ophthalmol. Strabismus.

[74] Ells A.L., Holmes J.M., Astle W.F., Williams G., A Leske D., Fielden M., Uphill B., Jennett P., Hebert M. (2003). Telemedicine approach to screening for severe retinopathy of prematurity. Ophthalmology.

[75] Quinn G.E., Ying G.S., Daniel E., Hildebrand P.L., Ells A., Baumritter A., Kemper A.R., Schron E.B., Wade K., for the e-ROP Cooperative Group (2014). Validity of a Telemedicine System for the Evaluation of Acute-Phase Retinopathy of Prematurity. JAMA Ophthalmol..

[76] Lorenz B., Spasovska K., Elflein H., Schneider N. (2009). Wide-field digital imaging-based telemedicine for screening for acute retinopathy of prematurity (ROP). Six-year results of a multicentre field study. Graefes Arch. Clin. Exp. Ophthalmol..

[77] Prakalapakorn S.G., Freedman S.F., Hutchinson A.K., Wallace D.K., Stinnett S.S., Riggins J.W., Gallaher K.J. (2018). Evaluating a Portable, Noncontact Fundus Camera for Retinopathy of Prematurity Screening by Nonophthalmologist Health Care Workers. Ophthalmol. Retin..

[78] NeoLight LLC ICON GO Portable Ophthalmic Retinal Imaging System. The NeoLight Company. https://theneolight.com/product-pages/icon-go.

[79] Derks L.A., Tekin Y.S., Loudon S.E., Vingerling J.R., Coyner A.S., Campbell J.P., Tjiam A.M. (2025). External validation of an artificial intelligence–based model for retinopathy of prematurity screening using Phoenix ICON retinal images. J. Am. Assoc. Pediatr. Ophthalmol. Strabismus.

[80] Teow Kheng Leong K., Abu Kassim S.N.A., Sidhu J.K., Sivalingam T., Ramasamy S., Khialdin S.M., Nordin N.M., Rahmat J. (2021). Neonatal eye screening for 203 healthy term new-borns using a wide-field digital retinal imaging system. BMC Ophthalmol..

[81] Vinekar A., Rao S.V., Murthy S., Jayadev C., Dogra M.R., Verma A., Shetty B. (2019). A Novel, Low-Cost, Wide-Field, Infant Retinal Camera, “Neo”: Technical and Safety Report for the Use on Premature Infants. Transl. Vis. Sci. Technol..

[82] Vinekar A., Bhende P. (2018). Innovations in technology and service delivery to improve Retinopathy of Prematurity care. Community Eye Health.

[83] Forus Health Pvt Ltd 3nethra neo—Neonatal ROP Screening & Wide-Field Imaging. https://forushealth.com/3nethra-neo/.

[84] Wood E.H., Moshfeghi A.A., Nudleman E.D., Moshfeghi D.M. (2016). Evaluation of Visunex Medical’s PanoCam^TM^ LT and PanoCam^TM^ Pro wide-field imaging systems for the screening of ROP in newborn infants. Expert Rev. Med. Devices.

[85] PanoCam^TM^ LT Wide-Field Digital Imaging System from Visunex Medical Systems. https://www.ophthalmologyweb.com/5818-Pediatric-Digital-Retinal-Imaging-Systems/9698287-PanoCam-LT-Wide-field-Digital-Imaging-System/.

[86] Karp K.A., Baumritter A., Pearson D.J., Pistilli M., Nyquist D., Huynh M., Satnes K., Keith R., Ying G.-S., Quinn G.E. (2016). Training retinal imagers for retinopathy of prematurity (ROP) screening. J. Am. Assoc. Pediatr. Ophthalmol. Strabismus.

[87] Yusuf A.M., Lusobya R.C., Mukisa J., Batte C., Nakanjako D., Juliet-Sengeri O. (2022). Validity of smartphone-based retinal photography (PEEK-retina) compared to the standard ophthalmic fundus camera in diagnosing diabetic retinopathy in Uganda: A cross-sectional study. PLoS ONE.

[88] Fierson W.M., Capone A., Granet D.B., Blocker R.J., Bradford G.E., Lehman S.S., Rubin S.E., The American Academy of Pediatrics Section on Ophthalmology, American Academy of Ophthalmology, American Association of Certified Orthoptists (2015). Telemedicine for Evaluation of Retinopathy of Prematurity. Pediatrics.

[89] Russo A., Morescalchi F., Costagliola C., Delcassi L., Semeraro F. (2015). A Novel Device to Exploit the Smartphone Camera for Fundus Photography. J. Ophthalmol..

[90] Morrison D., Bothun E.D., Ying G.S., Daniel E., Baumritter A., Quinn G. (2016). Impact of number and quality of retinal images in a telemedicine screening program for ROP: Results from the e-ROP study. J. Am. Assoc. Pediatr. Ophthalmol. Strabismus.

[91] Kovács G., Somogyvári Z., Maka E., Nagyjánosi L. (2017). Bedside ROP screening and telemedicine interpretation integrated to a neonatal transport system: Economic aspects and return on investment analysis. Early Hum. Dev..

[92] Skalet A.H., Quinn G.E., Ying G.S., Gordillo L., Dodobara L., Cocker K., Fielder A.R., Ells A.L., Mills M.D., Wilson C. (2008). Telemedicine screening for retinopathy of prematurity in developing countries using digital retinal images: A feasibility project. J. Am. Assoc. Pediatr. Ophthalmol. Strabismus.

[93] Sachdeva K., Turcios M., Solarte C. (2026). Pediatric teleophthalmology screening: A scoping review of global evidence and implications for Canada. Can. J. Ophthalmol..

[94] Almadhi N.H., Dow E.R., Paul Chan R.V., Alsulaiman S.M. (2022). Multimodal Imaging, Tele-Education, and Telemedicine in Retinopathy of Prematurity. Middle East Afr. J. Ophthalmol..

[95] Song A., Lee E., Ostmo S.R., Jonas K.E., Chervinko M.A., Coyner A.S., Campbell J.P., Chan R.P. (2026). Retinopathy of prematurity training and education: A systematic review. Surv. Ophthalmol..

[96] Patel S.N., Al-Khaled T., Kang K.B., Jonas M.K.E., Ostmo S., Ventura C.V., Martinez-Castellanos M.A., Anzures R.G.A.S., Campbell J.P., Chiang M.F. (2023). Characterization of Errors in Retinopathy of Prematurity Diagnosis by Ophthalmologists-in-Training in Middle-Income Countries. J. Pediatr. Ophthalmol. Strabismus.

[97] Patel S.N., Martinez-Castellanos M.A., Berrones-Medina D., Swan R., Ryan M.C., Jonas K.E., Ostmo S., Campbell J.P., Chiang M.F., Chan R.P. (2017). Assessment of a Tele-education System to Enhance Retinopathy of Prematurity Training by International Ophthalmologists-in-Training in Mexico. Ophthalmology.

[98] Ying G.S., VanderVeen D., Daniel E., Quinn G.E., Baumritter A. (2016). Risk Score for Predicting Treatment-Requiring Retinopathy of Prematurity (ROP) in the Telemedicine Approaches to Evaluating Acute-Phase ROP Study. Ophthalmology.

[99] Chiang M.F., Wang L., Busuioc M., Du Y.E., Chan P., Kane S.A., Lee T.C., Weissgold D.J., Berrocal A.M., Coki O. (2007). Telemedical Retinopathy of Prematurity Diagnosis: Accuracy, Reliability, and Image Quality. Arch. Ophthalmol..

[100] Xu C.L., Adu-Brimpong J., Moshfeghi H.P., Rosenblatt T.R., Yu M.D., Ji M.H., Wang S.K., Zaidi M., Ghoraba H., Michalak S. (2023). Telemedicine retinopathy of prematurity severity score (TeleROP-SS) versus modified activity score (mROP-ActS) retrospective comparison in SUNDROP cohort. Sci. Rep..

[101] Tsai A.S.H., Yip M., Song A., Tan G.S., Ting D.S., Campbell J.P., Coyner A., Chan R.V.P. (2024). Implementation of Artificial Intelligence in Retinopathy of Prematurity Care: Challenges and Opportunities. Int. Ophthalmol. Clin..

[102] Lin S., Ma Y., Xu Y., Lu L., He J., Zhu J., Peng Y., Yu T., Congdon N., Zou H. (2023). Artificial Intelligence in Community-Based Diabetic Retinopathy Telemedicine Screening in Urban China: Cost-effectiveness and Cost-Utility Analyses With Real-world Data. JMIR Public Health Surveill..

[103] Kim E., Nguyen K.H., Donovan T., Edirippulige S., Armfield N.R. (2023). A cost-minimisation analysis comparing alternative telemedicine screening approaches for retinopathy of prematurity. J. Telemed. Telecare.

[104] Kreofsky B.L.H., Blegen R.N., Lokken T.G., Kapraun S.M., Bushman M.S., Demaerschalk B.M. (2018). Sustainable Telemedicine: Designing and Building Infrastructure to Support a Comprehensive Telemedicine Practice. Telemed. E-Health.

[105] Al-Khaled T., Valikodath N.G., Patel S.N., Cole E., Chervinko M., Douglas C.E., Tsai A.S.H., Wu W.-C., Campbell J.P., Chiang M.F. (2021). Addressing the Third Epidemic of Retinopathy of Prematurity Through Telemedicine and Technology: A Systematic Review. J. Pediatr. Ophthalmol. Strabismus.

[106] Zhao X., Wu Z., Liu Y., Zhang H., Hu Y., Yuan D., Luo X., Zheng M., Yu Z., Ma D. (2024). Eyecare-cloud: An innovative electronic medical record cloud platform for pediatric research and clinical care. EPMA J..

[107] Braverman R.S., Jung J.L., Auer E.A., Patnaik J.L., Enzenauer R.W., Bhatia S.K., Siegel L.M., Robbins S.L. (2026). Tele-ROP care: The ophthalmologist perspective and experience. J. Am. Assoc. Pediatr. Ophthalmol. Strabismus.

[108] Luo Z., Ding X., Hou N., Wan J. (2022). A Deep-Learning-Based Collaborative Edge–Cloud Telemedicine System for Retinopathy of Prematurity. Sensors.

[109] Li J., Wilson L.S. (2013). Telehealth Trends and the Challenge for Infrastructure. Telemed. E-Health.

[110] Antonio-Aguirre B., Gadiraju A., Ownagh V., Vajzovic L. (2025). Novel artificial intelligence applications for pediatric retina. Curr. Opin. Ophthalmol..

[111] Vinekar A., Jayadev C., Mangalesh S., Shetty B., Vidyasagar D. (2015). Role of tele-medicine in retinopathy of prematurity screening in rural outreach centers in India—A report of 20,214 imaging sessions in the KIDROP program. Semin Fetal Neonatal Med..

[112] Vinekar A., Gilbert C., Dogra M., Kurian M., Shainesh G., Shetty B., Bauer N. (2014). The KIDROP model of combining strategies for providing retinopathy of prematurity screening in underserved areas in India using wide-field imaging, tele-medicine, non-physician graders and smart phone reporting. Indian J. Ophthalmol..

[113] Vinekar A., Jayadev C., Bauer N. (2015). Need for Telemedicine in Retinopathy of Prematurity in Middle-Income Countries: E-ROP vs KIDROP. JAMA Ophthalmol..

[114] Simkin S.K., Misra S.L., Han J.V., McGhee C.N.J., Dai S. (2019). Auckland regional telemedicine retinopathy of prematurity screening network: A 10-year review. Clin. Exp. Ophthalmol..

[115] Chan R.V.P., Patel S.N., Ryan M.C., Jonas K.E., Ostmo S., Port A.D., Sun G.I., Lauer A.K., Chiang M.F. (2015). The Global Education Network for Retinopathy of Prematurity (Gen-Rop): Development, Implementation, and Evaluation of A Novel Tele-Education System (An American Ophthalmological Society Thesis). Trans. Am. Ophthalmol. Soc..

[116] Chandwani R., Kumar N. (2018). Stitching Infrastructures to Facilitate Telemedicine for Low-Resource Environments. Proceedings of the 2018 CHI Conference on Human Factors in Computing Systems.

[117] Patel P.N., Patel P.A., Bhagat D., Chittaluru N., Bhatt H., Jager R., George M., Sheth V. (2022). Telemedicine for Retinal Disease During the COVID-19 Pandemic: Survey of the Patient Perspective. Ophthalmol. Ther..

[118] Das U., Cho E., Balasubramanian T., Zhou J., Mansoor S., Kapoor R., Forbes H.E., Chase S., Kore M., Sidelnikov D. (2025). Comparing infant pain and stress during retinopathy of prematurity screening using ophthalmoscopy and non-contact imaging. Sci. Rep..

[119] Dhami A., Gupta G., Dhami N.B., Arora N., Dhami G.S. (2021). Analysis of the parental satisfaction for retinopathy of prematurity screening using binocular indirect ophthalmoscopy versus wide field retinal imaging. Indian J. Ophthalmol..

[120] Horton M.B., Brady C.J., Cavallerano J., Abramoff M., Barker G., Chiang M.F., Crockett C.H., Garg S., Karth P., Liu Y. (2020). Practice Guidelines for Ocular Telehealth-Diabetic Retinopathy, Third Edition. Telemed. E-Health.

[121] Rahim S.A., Alshahrani S. (2023). Ethical Considerations in Telemedicine and Remote Healthcare. Saudi J. Nurs. Health Care.

[122] Solimini R., Busardò F.P., Gibelli F., Sirignano A., Ricci G. (2021). Ethical and Legal Challenges of Telemedicine in the Era of the COVID-19 Pandemic. Medicina.

[123] Rossi M., Rehman S. (2025). Integrating Artificial Intelligence Into Telemedicine: Evidence, Challenges, and Future Directions. Cureus.

[124] Bai A., Carty C., Dai S. (2022). Performance of deep-learning artificial intelligence algorithms in detecting retinopathy of prematurity: A systematic review. Saudi J. Ophthalmol..

[125] Bai A., Dai S., Hung J., Kirpalani A., Russell H., Elder J., Shah S., Carty C., Tan Z. (2023). Multicenter Validation of Deep Learning Algorithm ROP.AI for the Automated Diagnosis of Plus Disease in ROP. Transl. Vis. Sci. Technol..

[126] Tudor S., Bhatia R., Liem M., Wani T.A., Boyd J., Raza Khan U. (2025). Opportunities and Challenges of Using Artificial Intelligence in Predicting Clinical Outcomes and Length of Stay in Neonatal Intensive Care Units: Systematic Review. J. Med. Internet Res..

[127] Abbey A.M., Besirli C.G., Musch D.C., Andrews C.A., Capone A., Drenser K.A., Wallace D.K., Ostmo S., Chiang M., Lee P.P. (2016). Evaluation of Screening for Retinopathy of Prematurity by ROPtool or a Lay Reader. Ophthalmology.

[128] Ataer-Cansizoglu E., Bolon-Canedo V., Campbell J.P., Bozkurt A., Erdogmus D., Kalpathy-Cramer J., Patel S., Jonas K., Chan R.V.P., Ostmo S. (2015). Computer-Based Image Analysis for Plus Disease Diagnosis in Retinopathy of Prematurity: Performance of the “i-ROP” System and Image Features Associated With Expert Diagnosis. Transl. Vis. Sci. Technol..

[129] Brown J.M., Campbell J.P., Beers A., Chang K., Ostmo S., Chan R.V.P., Dy J., Erdogmus D., Ioannidis S., Kalpathy-Cramer J. (2018). Automated Diagnosis of Plus Disease in Retinopathy of Prematurity Using Deep Convolutional Neural Networks. JAMA Ophthalmol..

[130] Greenwald M.F., Danford I.D., Shahrawat M., Ostmo S., Brown J., Kalpathy-Cramer J., Bradshaw K., Schelonka R., Cohen H.S., Chan R.V.P. (2020). Evaluation of artificial intelligence-based telemedicine screening for retinopathy of prematurity. J. Am. Assoc. Pediatr. Ophthalmol. Strabismus.

[131] Patil J., Patil L., Parachuri N., Kumar N., Bandello F., Kuppermann B.D., Loewenstein A., Sharma A. (2020). Smartphone based ROP (S-ROP) screening—Opportunities and challenges. Eye.

[132] Chandrakanth P., Maitra P., Sivakumar P., Shah P.K., Narendran V. (2023). SMART (SMartphone-Assisted frugal Retinopathy of premaTurity screening). Indian J. Ophthalmol..

[133] Kim T.N., Myers F., Reber C., Loury P., Loumou P., Webster D., Echanique C., Li P., Davila J.R., Maamari R.N. (2018). A Smartphone-Based Tool for Rapid, Portable, and Automated Wide-Field Retinal Imaging. Transl. Vis. Sci. Technol..

[134] Shah D., Dewan L., Singh A., Jain D., Damani T., Pandit R., Porwal A.C., Bhatnagar S., Shrishrimal M., Patel A. (2021). Utility of a smartphone assisted direct ophthalmoscope camera for a general practitioner in screening of diabetic retinopathy at a primary health care center. Indian J. Ophthalmol..

[135] Ludwig C., Murthy S., Pappuru R., Jais A., Myung D., Chang R. (2016). A novel smartphone ophthalmic imaging adapter: User feasibility studies in Hyderabad, India. Indian J. Ophthalmol..

[136] Labkovich M., Paul M., Kim E., Serafini R.A., Lakhtakia S., A Valliani A., Warburton A.J., Patel A., Zhou D., Sklar B. (2022). Portable hardware & software technologies for addressing ophthalmic health disparities: A systematic review. Digit Health.

[137] Jin K., Li Y., Wu H., Tham Y.C., Koh V., Zhao Y., Kawasaki R., Grzybowski A., Ye J. (2024). Integration of smartphone technology and artificial intelligence for advanced ophthalmic care: A systematic review. Adv. Ophthalmol. Pract. Res..

[138] Adhikari S., Bajimaya S., Ranjitkar E.P. (2021). Validation of smartphone-based screening for retinopathy of prematurity in a low-resource setting. J. Am. Assoc. Pediatr. Ophthalmol. Strabismus.

[139] Sharma A., Goyal A., Bilong Y., Shah P., Banker A., Kumar N., Sharma R., Kuppermann B.D., Bandello F. (2019). Comparison of a Smartphone-Based Photography Method with Indirect Ophthalmoscopic Assessment in Referable Retinopathy of Prematurity. Ophthalmol. Retin..

[140] Thanos A., Yonekawa Y., Todorich B., Moshfeghi D.M., Trese M.T. (2016). Screening and treatments using telemedicine in retinopathy of prematurity. Eye Brain.

[141] Scruggs B.A., Chan R.V.P., Kalpathy-Cramer J., Chiang M.F., Campbell J.P. (2020). Artificial Intelligence in Retinopathy of Prematurity Diagnosis. Transl. Vis. Sci. Technol..

[142] Vinekar A., Jayadev C., Shetty B. (2016). Telemedicine in Retinopathy of Prematurity. Adv. Ophthalmol. Optom..

[143] Ahmed A., Shah S.V., Sedhom J., Brobby N.A.W., Zhao C.S., Overbey K., Deslauriers A., Appiah-Berko M.T., Aikins A., Tabin G. (2025). Pediatric Telemedicine-based Retinal Screening in Ghana. Niger. J. Vitreoretin Dis..

[144] Shah P.K., Appanraj R., Narendran V. (2018). Telemedicine for ROP. Asia-Pac. J. Ophthalmol..

